# Development of an Exercise Rehabilitation Functional Group for Individualized Exercise After Lumbar Spine Surgery

**DOI:** 10.3390/life15060943

**Published:** 2025-06-11

**Authors:** Seong Son, Han Byeol Park, Chan Jong Yoo, Moon-Hee Kim, Byung Rhae Yoo, Jae Ang Sim

**Affiliations:** 1 Department of Neurosurgery, Gil Medical Center, Gachon University College of Medicine, #24, 74th Street Namdongdaero, Namdong-Gu, Incheon 21565, Republic of Korea; sonseong@gilhospital.com (S.S.);; 2 Gachon Institute of Genome Medical and Science, Gachon University College of Medicine, Incheon 21565, Republic of Korea; 3 Department of Orthopedic Surgery, Gil Medical Center, Gachon University College of Medicine, Incheon 21565, Republic of Korea

**Keywords:** back muscles, exercise, low back pain, lumbar spine, postoperative care, rehabilitation

## Abstract

Individualized exercise therapy is crucial for effective postoperative rehabilitation. However, a widely accepted, standardized framework for measuring individual exercise capabilities after lumbar spine surgery (LSS) is lacking. This study aimed to develop a novel Exercise Rehabilitation Functional Group (ERFG) classification system to enable systematic, individualized rehabilitation after LSS. Eight exercise parameters and two clinical factors were assessed in 428 volunteers within 12 weeks of single-segment LSS to establish reference data for the ERFG. The study cohort included 411 participants (mean age 67.10 ± 11.60; 128 males and 283 females) with an average postoperative interval of 42.96 ± 20.25 days. Key metrics included lumbar spine ROM (mean 74.24 ± 25.30°), trunk muscle strength (mean 15.71 ± 5.14 kg), trunk muscle endurance (mean 95.80 ± 35.66 s), whole-body flexibility (mean 3.30 ± 10.65 cm), cardiopulmonary capability (mean 433.91 ± 118.75 m), gait with affected single leg (mean 36.26 ± 5.98%), single-leg balance (median 13.60 s), coordination capability (mean 8.21 ± 3.23 s), EuroQol 5-Dimension 5-Level score (mean 9.39 ± 4.17), and visual analog scale for low back pain (mean 3.78 ± 2.55). Data were categorized into five grades using Cajori’s five-grade mathematical method. Significant correlations were observed between the eight exercise parameters and two clinical factors. This study established a foundational framework for standardizing baseline exercise capabilities after LSS. This ERFG system may provide a basis for individualized rehabilitation strategies to enhance patient outcomes.

## 1. Introduction

Lumbar spine surgery is a common intervention for various spinal disorders with the aim of alleviating pain, restoring function, and improving quality of life. Postoperative rehabilitation is crucial for optimizing recovery, preventing complications, and enhancing functional outcomes [1]. Functional exercise programs, which focus on improving strength, flexibility, and overall functionality, play a vital role in the rehabilitation process [2,3,4,5]. Additionally, early exercise therapy after lumbar spine surgery has been associated with enhanced recovery, shorter hospital stays, reduced opioid consumption, and improved postoperative outcomes, addressing concerns related to rising opioid use and prolonged hospitalization [6,7].

Although a potential link exists among trunk core muscles, kinematic movement, and low back pain [8,9], no widely accepted standardized framework exists for measuring individual exercise capabilities after lumbar spinal surgery. Traditional subjective methods for assessing exercise capability are prone to variability and bias, potentially leading to inconsistent rehabilitation outcomes [4,10]. An accurate and objective assessment of patient performance is essential for designing exercise programs and monitoring outcomes [11]. An objective grading system offers a structured and quantifiable approach to measuring performance, enabling tailored rehabilitation strategies with greater precision [12].

This study aimed to develop and implement an objective grading system, the Exercise Rehabilitation Functional Group (ERFG), to scientifically evaluate functional capacity in patients after lumbar spine surgery. The system evaluates key parameters to tailor individualized rehabilitation programs and monitors treatment outcomes. Standardizing the evaluation process ensures that patients receive the most effective rehabilitation, ultimately leading to improved long-term outcomes and quality of life.

## 2. Materials and Methods

### 2.1. Trial Design and Ethics

This single-center, single-arm, open-label trial was performed in accordance with the 1964 Declaration of Helsinki and subsequent amendments. Institutional Review Board approval was obtained on 14 February 2022 (GDIRB2022-095). This study was registered with the Clinical Research Information Service of the Republic of Korea (http://cris.nih.go.kr (accessed on 13 February 2022), number KCT0006880).

### 2.2. Study Aims

The primary aim of this study was to evaluate individual exercise and functional capacity and to establish an ERFG system in patients who underwent lumbar spine surgery. The primary endpoint was to analyze and categorize all data into appropriate groups based on statistical distribution. The secondary endpoint was the correlation between parameters.

### 2.3. Sample Size

The sample size was calculated using the following formula:

$$n={\left(z_{\alpha /2}\frac{\sigma }{d}\right)}^{2}$$

The standard deviation (*σ*) for the exam was set to 5.0, and the margin of error (*d*) was set to 0.5. With a significance level of 5% and a confidence level of 95%, the required sample size was 385. Accounting for a 10% dropout rate, a total of 428 participants were recruited.

### 2.4. Participant Recruitment

Participants were recruited from outpatient and inpatient clinics by four neurosurgeons specializing in lumbar spine surgery based on inclusion and exclusion criteria.

The study included (1) adults aged 20–70 years within 6 weeks after single-segment lumbar spine surgery (from L1 to S1) without adverse events, such as surgical site infection or recurrence; (2) patients who underwent surgery, including discectomy, decompression, or fusion, for lumbar degenerative diseases, including lumbar disc herniation, spinal stenosis, or spondylolisthesis; (3) patients who could ambulate sufficiently to perform an assessment of exercise capacity; and (4) patients who voluntarily decided to participate in the trial.

The study excluded (1) those who could not perform an assessment of exercise capacity, such as those with paralysis (motor grade ≤ 3), bladder or bowel dysfunction, concurrent lower-extremity disease, or extreme pain making daily activities impossible; (2) patients who were unable to understand consent or had difficulty performing the exercise movements independently (e.g., patients with mental disorders or reduced cognitive function); and (3) those deemed ineligible to participate in this trial based on the researcher’s judgment.

### 2.5. Time Frame

The study was conducted over 1.5 years. Informed consent was obtained from all participants before registration. The participants received a 1 h education to follow the correct measuring performance prior to the official assessment for accurate measurement. A detailed assessment of exercise capacity was conducted on the same day to ensure consistency.

### 2.6. ERFG Parameters and Assessment

Two certified exercise therapists and one investigator conducted the exercise education, assessment, and data collection. Baseline data included age, sex, interval from surgery to assessment, height, weight, and body mass index (BMI). Body proportion analysis, including body mass index, skeletal muscle mass, total body fat mass, and total body fat percentage, was also investigated using Inbody^®^ (Inbody, Seoul, Republic of Korea).

ERFG parameters included the following: (1) range of motion of the lumbar spine, (2) trunk muscle strength, (3) trunk muscle endurance, (4) flexibility of the whole body, (5) cardiorespiratory capability, (6) gait pattern, (7) single-leg balance, (8) coordination capability, (9) quality of life, and (10) degree of pain.

The range of motion of the lumbar spine (°), measured as the difference between the degree of full flexion and full extension angle in the straight standing position, was defined as the average of six measured recordings using the digital goniometer HALO© (HALO Medical Devices, Sydney, Australia) [13,14,15].

Trunk muscle strength (kg), that is, the mean strength on the sternal notch during maximal flexion and strength on the C7-T1 spinous process during full extension for 3 s in the sitting position, was defined as the average of three measurements of strength using a digital handheld dynamometer (Micro FET2^®^, Seed technology, Bucheon, Republic of Korea) [16].

Trunk muscle endurance (seconds), defined as the mean duration of maintaining 50% of each subject’s strength in maximum flexion and extension in the sitting position, was evaluated using a digital timer and dynamometer [17].

The flexibility of the whole body (cm), that is, the distance between both fingertips and toes when the upper body was bent forward for 3 s in a position on the floor with the legs stretched (Sit-and-Reach Test), was defined as the average of two measurements [18].

Cardiopulmonary capability (m), measured as the distance covered during a 6 min fast walk (6 min walk test), was assessed once [19].

Gait pattern (%), defined as the percentage of unilateral major foot support time during the 6 min fast walk, was analyzed using the Neurogait^®^ app (Salted, Hanam, Republic of Korea) [20].

General balance (s), measured as the maximum time to stand on one foot (Single-Leg Stance test), was evaluated by averaging three measurements [21].

Coordination capability (seconds), defined as the time spent standing from sitting in a chair, walking 6 m, and returning to the chair (the Timed Up-And-Go test), was derived as the average of two measurements [22,23].

Quality of life was assessed based on the EuroQol 5-Dimension 5-Level version (EQ-5D-5L), ranging from 5 to 25 points [24].

The degree of pain was evaluated based on a visual analog scale (VAS) ranging from 0 to 10 points [25].

The parameters were divided into five groups based on their positions in the standard distribution using Cajori’s five-grade mathematical method [26].

### 2.7. Statistical Analysis

Quantitative analysis of the data was conducted by a specialist doctor and a statistician who was blinded to the participants’ information. Data management and statistical analyses were conducted using SPSS (version 27.0; IBM Corporation, Armonk, NY, USA).

The normal distribution of the data was evaluated using the Kolmogorov–Smirnov test and Shapiro–Wilk test, and all data are reported as mean ± standard deviation or median and interquartile range (IQR), depending on the distribution of the data. Pearson’s correlation test or the Spearman correlation test was used to evaluate the correlation between the parameters. Statistical significance was set at *p* < 0.05.

## 3. Results

### 3.1. Baseline Characteristics

**Figure 1:**
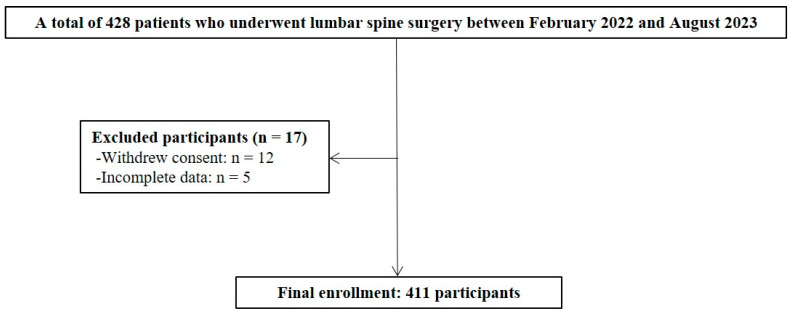
Flow diagram showing patient recruitment.

Of the 428 participants initially recruited, 12 dropped out due to measurement failure and/or withdrawal of consent during education and assessment, and five were excluded due to lack of data or incomplete data (Figure 1). The final cohort included 411 participants (129 men and 282 women) with a mean age of 67.22 ± 11.55 years. The baseline characteristics of the patients are described in Table 1.

### 3.2. Overall Results of Parameters and ERFG Categorization

All parameters, except single-leg balance, showed a normal distribution. These metrics are listed (Table 2).

(1) Mean range of motion of the lumbar spine: 74.24 ± 25.30°;
(2) Mean strength of flex/extension: 15.71 ± 5.14 kg;
(3) Mean endurance of flexion/extension: 95.80 ± 35.66 s;
(4) Mean flexibility of the whole body: 3.30 ± 10.65 cm;
(5) Mean cardiopulmonary capability: 433.91 ± 118.75 m;
(6) Mean gait with affected single leg: 36.26 ± 5.98%;
(7) Median single-leg balance of affected side: 13.60 (IQR, 3.02–32.00) seconds;
(8) Mean coordination capability: 8.21 ± 3.23 s;
(9) Mean EuroQol 5-Dimension 5-Level version: 9.39 ± 4.17,
(10) Mean visual analog scale for low back pain: 3.78 ± 2.55.

Each parameter was divided into five groups based on sex classification using the Cajori five-grade method (Table 3).

### 3.3. Correlation Between Parameters

Most of the parameters demonstrated significant correlations with each other. Notably, clinical parameters such as the EQ-5D-5L and VAS scores were significantly correlated with all exercise parameters, as determined by Pearson’s correlation test or Spearman correlation tests (Table 4).

### 3.4. Safety and Side Effects

No adverse events or side effects were reported during the study.

## 4. Discussion

Exercise rehabilitation therapy has been established as clinically beneficial for patients with chronic low back pain and those recovering from spinal surgery [27,28]. Specifically, exercises aimed at improving range of motion, flexibility, and strengthening core and extensor muscles have been shown to significantly alleviate pain and enhance quality of life [11,29,30,31]. However, a universally applicable exercise therapy protocol has yet to be developed, and there remains no consensus on objective measurement and follow-up methods for evaluating exercise capacity. Therefore, the development of a standardized measurement and grading system is critical for the systematic application and evaluation of rehabilitation exercise therapy in patients after spinal surgery.

We believe that there must be an objective evaluation system that assesses the patient’s functional capacity to apply individualized exercise rehabilitation and monitor the outcome of exercise treatment after lumbar spine surgery. We developed an ERFG system for the scientific evaluation of exercise ability. Subsequently, we investigated the functional capacity of a large number of participants who underwent lumbar spine surgery in detail and established reference data.

In this study, we focused on eight key parameters for measuring lumbar exercise capacity: range of motion of the lumbar spine [32,33,34], trunk muscle strength [35], trunk muscle endurance [36], whole-body flexibility [37,38], cardiopulmonary capacity (6 min walk test) [39,40], gait pattern [41], single-leg balance (Single-Leg Stance test) [42,43,44], and coordination capability (the Timed Up-and-Go test) [45,46]. These parameters were selected based on their established utility in assessing exercise capacity in patients with lumbar spine conditions and their ability to be quantitatively and objectively measured [32,35,36,37,39,41,42,45]. Additionally, these parameters are known to be significantly related to clinical outcomes such as low back pain and quality of life [11,33,34,36,38,40,43,44,46].

Our findings demonstrate that these parameters exhibit gradable distributions in post-spinal surgery patients, allowing for successful classification using a standardized grading method. The five-stage grading system developed in this study offers a practical and systematic approach to defining individual exercise capacity, enabling the application of tailored exercise therapy. Without an objective assessment of the patient’s current exercise capacity, there is a risk of prescribing either excessive or insufficient exercise, which limits the efficacy of rehabilitation therapy. In contrast, the proposed grading system allows the design of individual tailored postoperative exercise. For instance, patients classified as Level II in a specific exercise parameter may receive tailored exercise prescriptions to progress to Levels III or IV. This approach facilitates personalized exercise therapy, supports incremental improvements in clinical outcomes, and provides a framework for the objective monitoring of progress.

Although most of the eight parameters of exercise showed significant correlations with each other, some demonstrated an independent pattern. Furthermore, as supported by the existing literature and clinical observations, all measured exercise capacity parameters showed statistically significant correlations with low back pain and quality of life. This indicates that each objective measurement of exercise parameters has direct clinical relevance and implications for patient outcomes. Accordingly, individualized assessment and training of each parameter are valuable for enhancing clinical outcomes and supporting a multifaceted rehabilitation approach.

This study has several limitations. First, the sample size, which was sufficient for the initial analyses, may not have been sufficiently large to establish universally standardized values. Second, the lack of age-based stratification precludes the development of age-specific standards. Third, we were unable to account for the type and specific time interval between surgery and assessment, which restricted our ability to tailor parameter measurements according to surgical variables. Additionally, while the study incorporated sex-based grading, it did not account for other factors such as body mass index, muscle mass, underlying comorbidities, lifestyle patterns, or ethnic diversity. These variables are likely to influence exercise capacity and should be considered in future studies to achieve a more robust standardization.

Nonetheless, this study is significant as it is the first to measure and quantify representative exercise capacity parameters related to the lumbar spine in post-surgical patients. This study provides a foundation for the systematic measurement, prescription, and monitoring of customized exercise therapy in this population. Future studies should aim to expand the sample size, include broader demographic variables, and refine the grading system to improve its generalizability and applicability across diverse patient populations.

## 5. Conclusions

This study established a comprehensive dataset and grading system to define the baseline exercise capacity in patients after lumbar spine surgery. The ERFG system provides a valuable framework for implementing patient-tailored exercise therapy and scientifically evaluating performance. Furthermore, this study provides foundational data to guide future research on exercise rehabilitation strategies for patients after lumbar surgery.

## Figures and Tables

**Table 1:** Baseline characteristics.

Variable	Value
Mean age, years	67.22 ± 11.55
Male:Female	129:282
Interval from surgery, days	21.48 ± 15.23
Height, cm	160.50 ± 8.76
Weight, kg	64.09 ± 18.06
Body mass index, kg/m^2^	24.56 ± 5.44
Skeletal muscle mass, kg	23.88 ± 15.15
Total body fat mass, kg	20.15 ± 7.25
Total body fat percentage, %	30.53 ± 9.68
Alcohol, yes (%)	117 (28.5%)
Smoking, yes (%)	70 (17.0%)
Baseline status of exercise, none:irregular:regular (%)	151 (36.7%):157 (38.2%):103 (25.1%)

**Table 2:** Overall results of parameters.

Variable	Value
Range of motion of the lumbar spine, °	74.24 ± 25.30
Trunk muscle strength, kg	
Flexion	14.04 ± 4.89
Extension	17.38 ± 5.92
Mean	15.71 ± 5.14
Trunk muscle endurance, seconds	42.95 ± 30.26
Flexion	94.86 ± 37.80
Extension	96.75 ± 36.00
Mean	95.80 ± 35.66
Flexibility of the whole body, cm	3.30 ± 10.65
Cardiopulmonary capability, m	433.91 ± 118.75
Gait pattern, %	
Affected side	36.26 ± 5.98
Healthy side	39.53 ± 5.15
Mean	37.90 ± 5.57
Single-leg balance, seconds	
Affected side	13.60 (IQR, 3.02–32.00)
Healthy side	8.58 (IQR, 4.00–41.81)
Mean	10.42 (IQR, 3.74–37.31)
Coordination capability, seconds	8.21 ± 3.23
Quality of life (EQ-5D-5L)	9.39 ± 4.17
Degree of pain (VAS)	3.78 ± 2.55

EQ-5D-5L, EuroQol 5-Dimension 5-Level version; IQR, interquartile range; VAS, visual analog scale.

**Table 3:** Exercise rehabilitation functional group.

Variable		1st Grade (Very Poor)	2nd Grade (Poor)	3rd Grade (Average)	4th Grade (Good)	5th Grade (Very Good)
Range of motion of the lumbar spine, °						
	Male	≤48.5	48.6–58.6	58.7–92.2	92.3–115.4	≥115.5
	Female	≤40.9	40.8–53.9	54.0–89.2	89.3–111.9	≥112.0
Trunk muscle strength, kg						
	Male	≤13.1	13.2–16.1	16.2–22.2	22.3–24.7	≥24.8
	Female	≤8.2	8.3–10.6	10.7–12.2	12.3–17.3	≥17.4
Trunk muscle endurance, seconds						
	Male	≤25.2	25.3–31.6	31.7–76.0	76.1–119.9	≥120.0
	Female	≤19.9	20.0–25.8	25.9–74.1	74.2–119.9	≥120.0
Flexibility of the whole body, cm						
	Male	≤−13.0	−12.9–−8.0	−8.1–4.6	4.7–10.3	≥10.4
	Female	≤−11.7	−11.6–−0.8	−0.9–13.2	13.3–19.2	≥19.3
Cardiorespiratory capability, m						
	Male	≤356.2	376.3–411.2	411.3–539.9	540.0–601.9	≥602
	Female	≤272.7	272.8–359.9	360.0–493.1	493.2–553.2	≥553.3
Gait pattern, %						
	Male	≤32.0	32.1–36.8	36.9–40.5	40.6–42.2	≥42.3
	Female	≤31.9	42.0–36.4	36.5–40.1	40.2–47.2	≥47.3
Single-leg balance, seconds						
	Male	≤2.1	2.2–3.9	4.0–42.6	42.7–67.2	≥67.3
	Female	≤1.9	2.0–3.7	3.8–36.7	36.8–84.0	≥84.1
Coordination capability, seconds						
	Male	≤5.4	5.5–6.0	6.1–8.0	8.1–9.0	≥9.1
	Female	≤5.0	5.1–6.9	7.0–11.9	12.0–13.9	≥14.0
Quality of life (EQ-5D-5L)		≥16	12–15	6–11	4–5	≤3
Degree of pain (VAS)		≥8	6–7	3–5	1–2	0

EQ-5D-5L, EuroQol 5-Dimension 5-Level version; VAS, visual analog scale.

**Table 4:** Correlation between all parameters.

Variable	Range of Motion of the Lumbar Spine	Trunk Muscle Strength	Trunk Muscle Endurance	Flexibility of the Whole Body	Cardiorespiratory Capability	Gait Pattern	Single-Leg Balance	Coordination Capability	Quality of Life (EQ-5D-5L)	Degree of Pain (VAS)
Range of motion of the lumbar spine		0.264	0.070	0.246	0.089	0.231	−0.333	0.202	−0.202	−0.142
		<0.001	0.159	<0.001	<0.001	0.086	<0.001	<0.001	<0.001	0.004
Trunk muscle strength			0.289	−0.025	0.453	0.138	0.335	−0.403	−0.200	−0.400
			<0.001	0.629	<0.001	0.008	<0.001	<0.001	<0.001	<0.001
Trunk muscle endurance				0.132	0.238	0.015	0.222	−0.305	−0.172	−0.247
				0.010	<0.001	0.772	<0.001	<0.001	<0.001	<0.001
Flexibility of the whole body					0.106	0.017	−0.077	−0.106	−0.107	−0.006
					0.037	0.746	0.133	0.037	0.036	0.903
Cardiorespiratory capability						0.245	0.514	−0.728	−0.289	−0.244
						<0.001	<0.001	<0.001	<0.001	<0.001
Gait pattern							0.152	−0.264	−0.034	−0.104
							0.004	<0.001	0.477	0.047
Single-leg balance								−0.431	−0.188	−0.216
								<0.001	<0.001	<0.001
Coordination capability									0.279	0.181
									<0.001	<0.001
Quality of life (EQ-5D-5L)										0.430
										<0.001
Degree of pain (VAS)										

EQ-5D-5L, EuroQol 5-Dimension 5-Level version; VAS, visual analog scale.

## Data Availability

The data presented in this study are available on request from the corresponding author. The data are not publicly available due to patient privacy concerns.

## Abbreviations

EQ-5D-5L	EuroQol-5 Dimension 5-Level version
IQR	interquartile range
VAS	visual analog scale
